# Supplementary material for: Proteostasis by STUB1/HSP70 complex controls sensitivity to androgen receptor targeted therapy in advanced prostate cancer
Source: Nat Commun. 2018 Nov 16;9:4700. doi: 10.1038/s41467-018-07178-x (PMC6240084; doi:10.1038/s41467-018-07178-x)
Supplement: Supplementary file 1 — Supplementary Information [file 41467_2018_7178_MOESM1_ESM.pdf]

Supplementary Information

Proteostasis by STUB1/HSP70 complex controls sensitivity to androgen receptor targeted therapy in advanced prostate cancer

Liu, et al.

Supplementary Figure 1

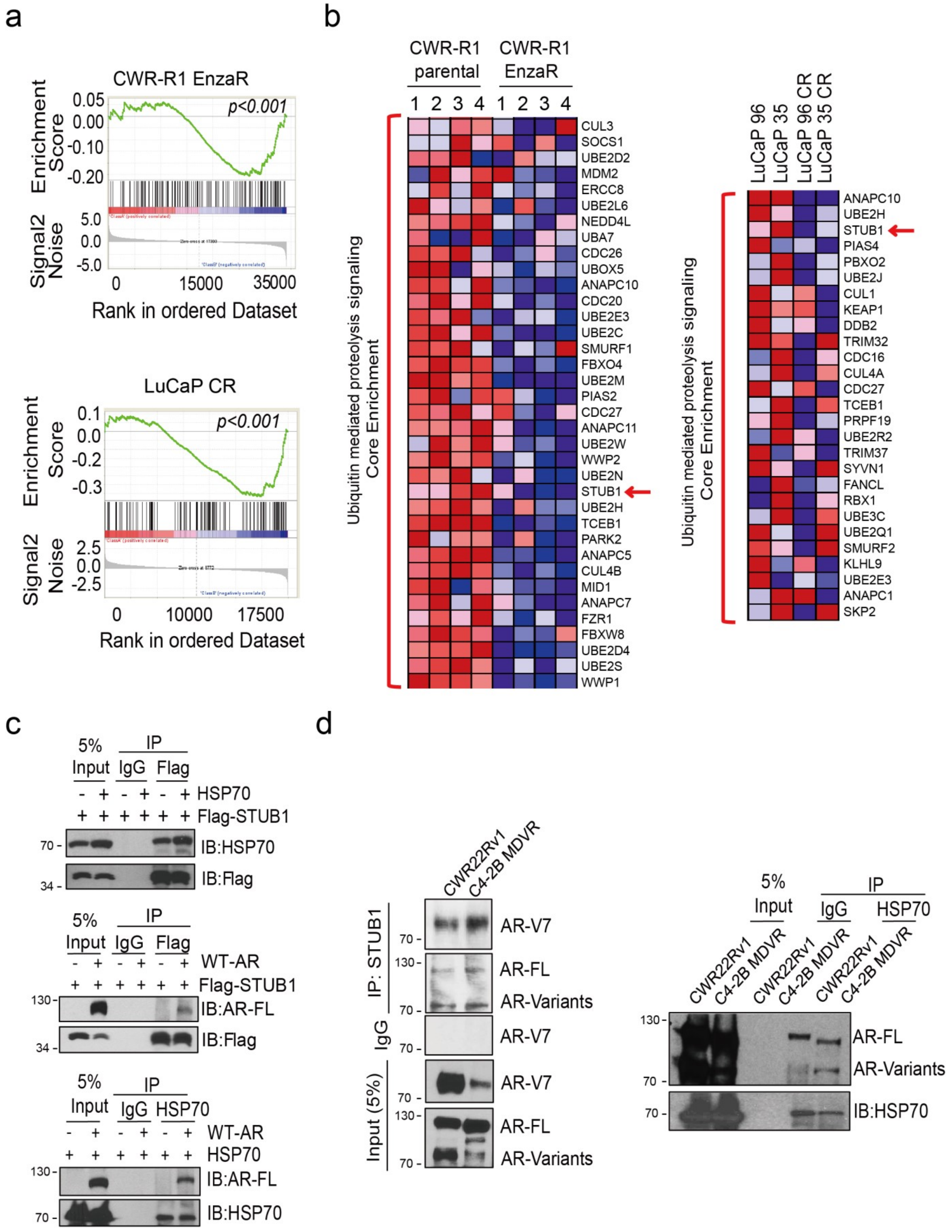

Supplementary Figure 1. The UPS is altered in enzalutamide resistant prostate cancer. **(a)** Global gene microarray data from CWR-R1 parental and CWR-R1 EnzaR cells, LuCaP and LuCaP CR tumors were analyzed by GSEA, ubiquitin mediated proteolysis gene set was enriched in resistant cells and LuCaP CR models. **(b)** Core-enrichment genes of ubiquitin mediated proteolysis pathway in CWR-R1 EnzaR cells and LuCaP CR tumors. The red arrow showed STUB1 was suppressed in resistant cells and LuCaP CR tumors. **(c)** 293 cells were transiently transfected with Flag-STUB1 or HSP70 with or without WT-AR for 3 days and whole cell lysates were immunopreciptated with anti-Flag or HSP70 antibodies and blotted with AR, Flag or HSP70 antibodies. **(d)** Whole cell lysates from C4-2B MDVR and CWR22Rv1 cells were extracted and immunopreciptated with anti-STUB1 (left) or HSP70 (right) antibodies and blotted with AR-V7 and AR antibodies.

Supplementary Figure 2

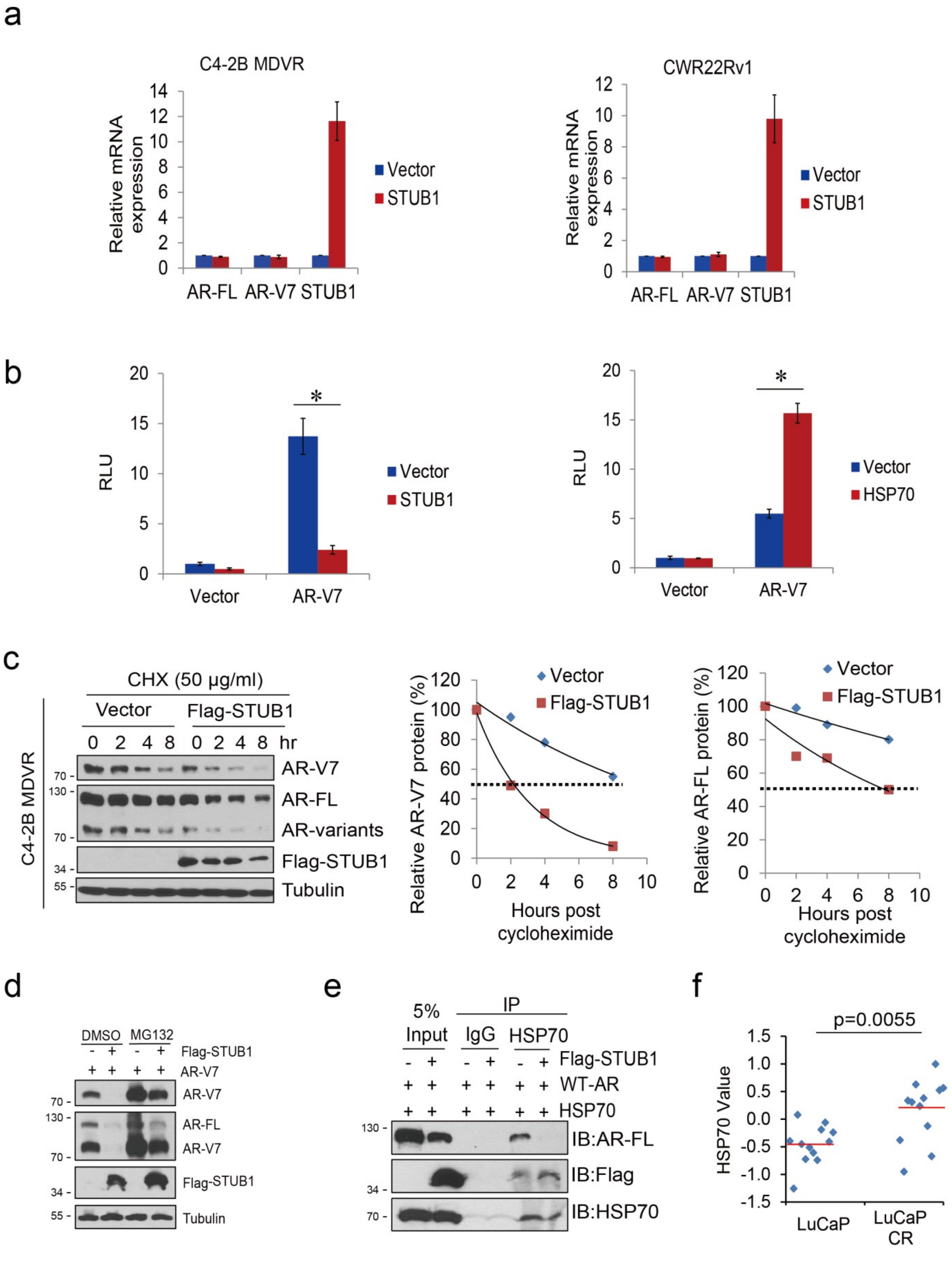

Supplementary Figure 2. STUB1 regulates AR-V7 expression and activity. (a) CWR22Rv1 and C4-2B MDVR cells were transiently transfected with 2 µg or 5 µg Flag-STUB1 for 3 days, total RNA was extracted and mRNA expression of AR-FL, AR-V7 and STUB1 was determined by qRT-PCR. **(b)** C4-2B cells were transiently transfected with vector plasmid, AR-V7 and PSA-luciferase plasmid with or without STUB1 (left) or HSP70 (right) plasmid, the PSA luciferase activity was determined 3 days after transfection. **(c)** C4-2B MDVR cells were transiently transfected with vector or flag-STUB1 and then treated with 50 µg per mL cycloheximide (CHX), total cell lysates were collected at 0, 2, 4 and 8 hours after the treatment and subjected to western blot and the half-life of AR-V7 and AR-FL were calculated. **(d)** C4-2B cells were transiently transfected with AR-V7 with or without STUB1 for 3 days and then treated with 5 µM MG132 overnight. Total cell lysates were collected and subjected to western blot. **(e)** 293 cells were co-transfected with HSP70, AR-FL with or without Flag-STUB1 for 3 days, whole cell lysates were immunopreciptated with anti-HSP70 antibody and blotted with AR, HSP70 and Flag antibodies. **(f)** Gene expression data was extracted from GSE93812 data set and HSP70 expression was determined in LuCaP parental and LuCaP CR PDX tumor samples. * $p<0.05$. Results are the mean of three independent experiments (±S.D.). Statistical analysis was performed using two tailed Student's t test.

Supplementary Figure 3

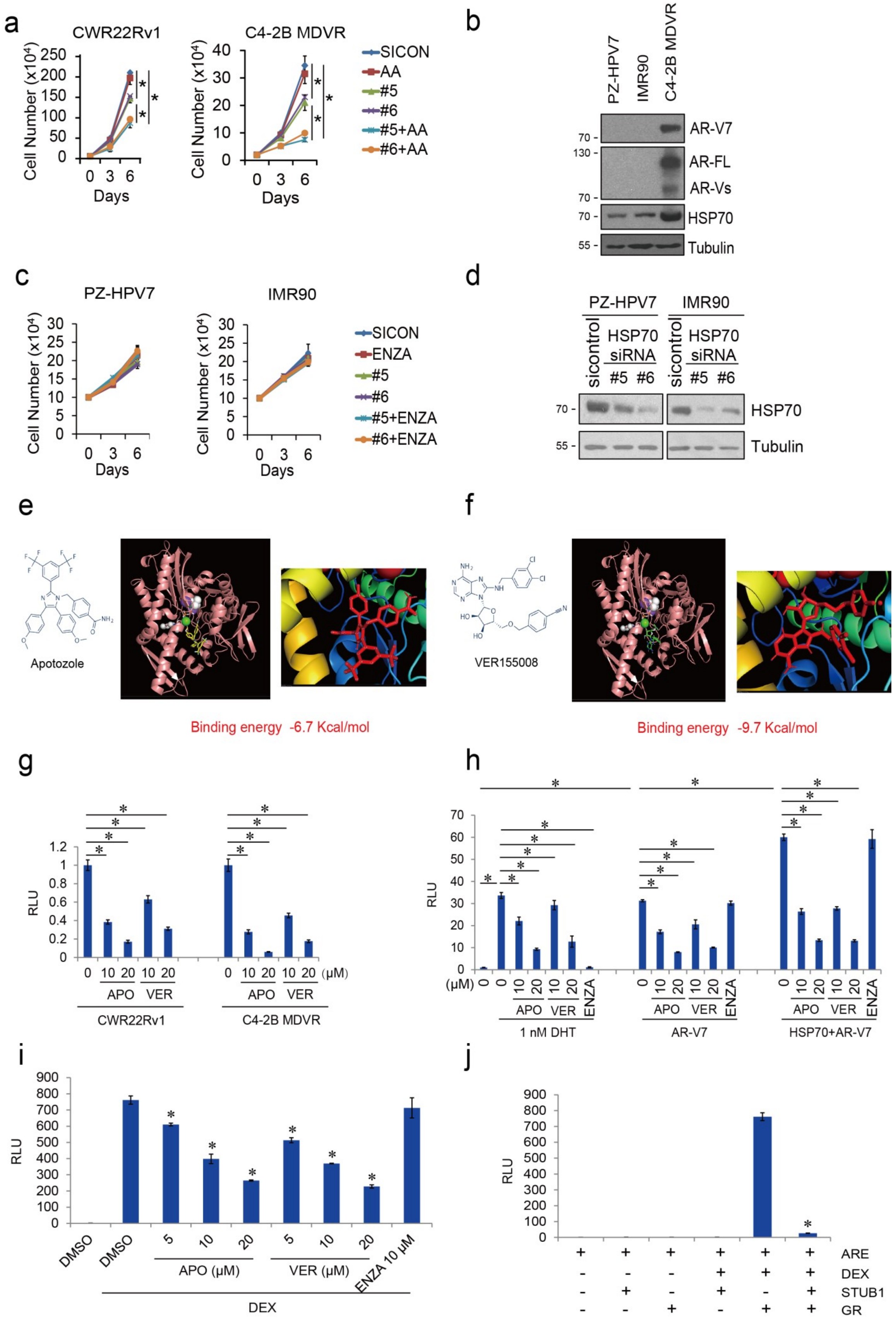

Supplementary Figure 3. HSP70 inhibition suppresses AR-V7 transcriptional activity and prostate cancer cell growth. (a) CWR22Rv1 and C4-2B MDVR cells were transiently transfected with two independent HSP70 siRNA (#5 and #6) and then treated with 5 μM abiraterone. Total cell numbers was determined at 3 and 6 days. **(b)** Whole cell lysates from PZ-HPV7, IMR90 and C4-2B MDVR cells were extracted and subjected to western blot. **(c-d)** PZ-HPV7 and IMR90 cells were transiently transfected with two independent HSP70 siRNA (#5 and #6) and then treated with 20 μM enzalutamide. Total cell number was determined at 3 and 6 days. Whole cell lysates were collected and subjected to western blot. **(e)** Structure of APO (left). Docking model of APO and HSP70 ATPase pocket, the lowest binding energy was -6.7 Kcal per mol (right) through AutoDock vina and PyMOL. **(f)** Structure of VER (left). Docking model of VER and HSP70 ATPase binding pocket, the lowest binding energy was -9.7 Kcal per mol (right) through AutoDock vina and PyMOL. **(g)** CWR22Rv1 and C4-2B MDVR cells were transiently transfected with PSA luciferase plasmid and then treated with different concentrations of APO or VER overnight, the PSA luciferase activity was determined. **(h)** C4-2B cells were transiently transfected with vector, AR-V7 or HSP70 plus AR-V7, treated with 1 nM DHT or control and then treated with different concentrations of HSP70 inhibitors or 20 μM enzalutamide overnight. PSA luciferase activity was determined. **(i)** 293 cells were transiently transfected with vector or GR, treated with 10 nM DEX or DMSO and then treated with different concentrations of HSP70 inhibitors or 10 μM enzalutamide overnight. ARE luciferase activity was determined. **(j)** 293 cells were transiently transfected with vector, GR or STUB1 for 3 days and treated with 10 nM DEX or DMSO overnight. ARE luciferase activity was determined. * $p<0.05$ Results are the mean of three independent experiments (±S.D.). Statistical analysis was performed using one way ANOVA. ARE: Androgen response element, DEX: Dexamethasone. GR: Glucocorticoid receptor. APO: Apoptozole, VER: Ver155008.

Supplementary Figure 4

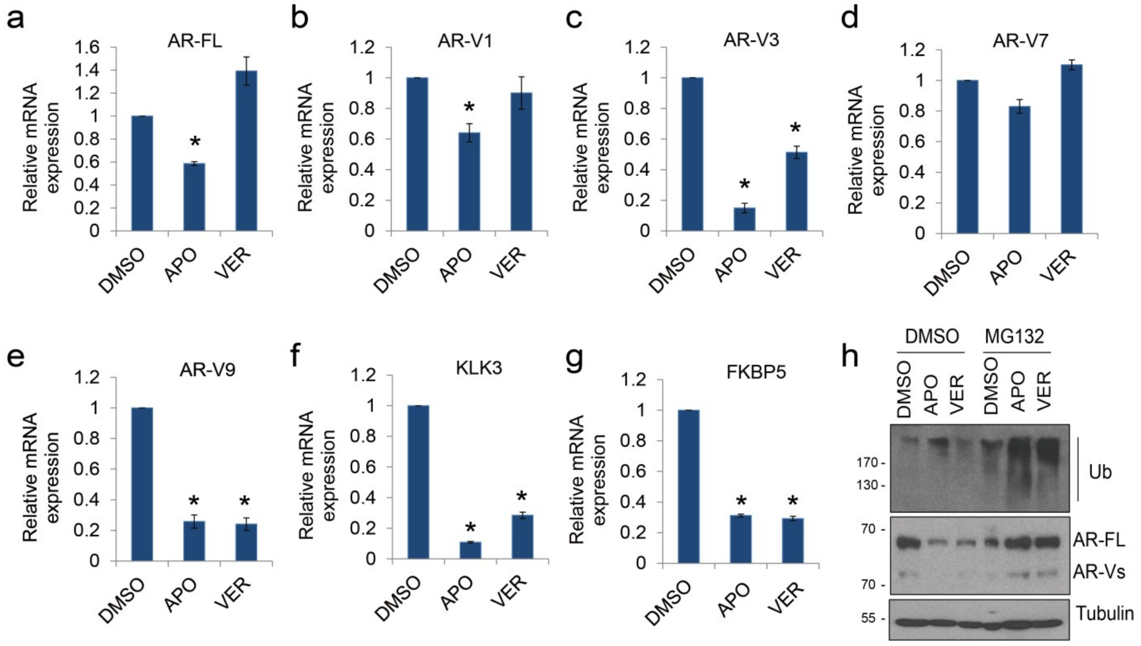

Supplementary Figure 4. HSP70 inhibitors affect AR/AR variants mRNA expression. (a-g) C4-2B MDVR cells were treated with 20 μM APO or VER for 24 hours, total RNA was extracted from the cells and mRNA levels of AR-FL, AR-V1, AR-V3, AR-V7, AR-V9, KLK3 and FKBP5 was determined by qRT-PCR. (**h**) C4-2B MDVR cells were treated with 20 μM APO or VER for 24 hours, followed by 5 μM MG132 for additional 6 hours, whole cell lysates were extracted and subjected to western blot. * $p<0.05$. Results are the mean of three independent experiments (±S.D.). Statistical analysis was performed using two tailed Student's t test. APO: Apoptozole, VER: Ver155008.

Supplementary Figure 5

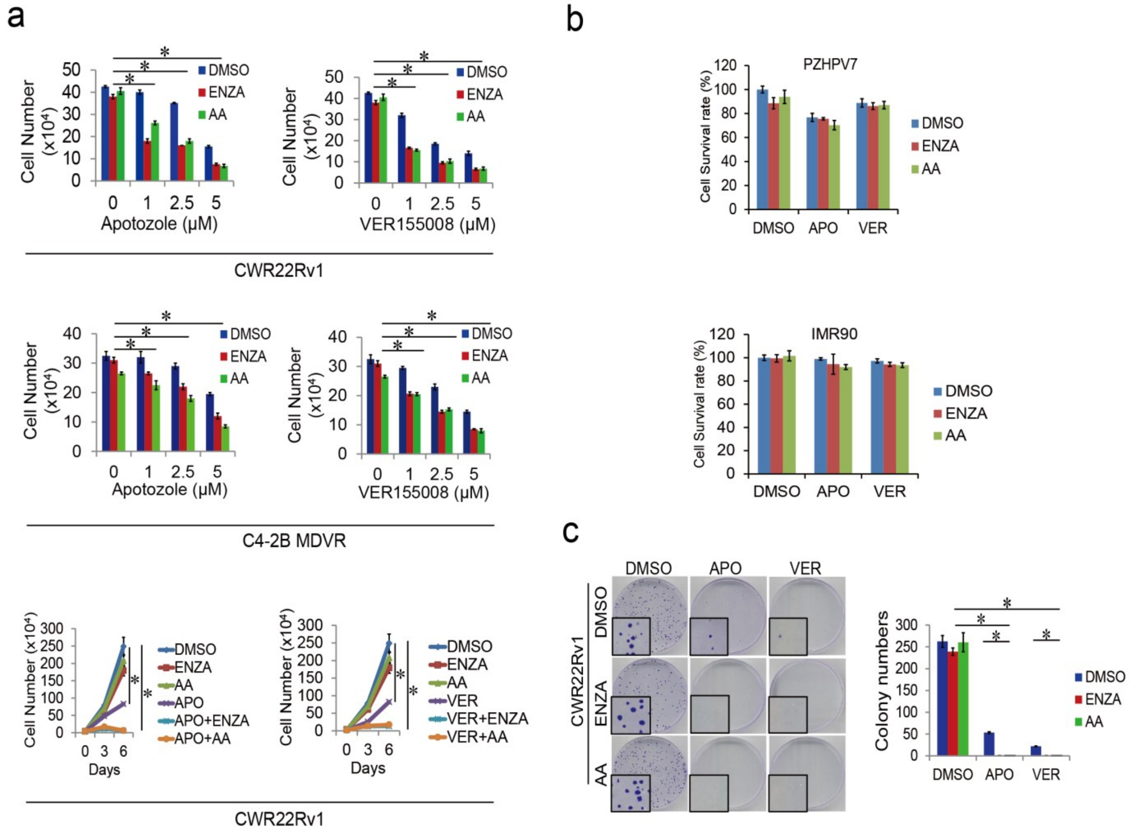

Supplementary Figure 5. HSP70 inhibitors enhance Enza/AA treatment. (a) CWR22Rv1 and C4-2B MDVR cells were treated with 1, 2.5 and 5 µM APO or VER with or without 20 µM enzalutamide or 5 µM abiraterone, total cell number was determined at 3 days (top and middle). CWR22Rv1 cells were treated with 5 µM APO or VER with or without enzalutamide and abiraterone, total cell number was determined at 3 and 6 days (bottom). (**b**) PZ-HPV7 and IMR90 cells were treated with 5 µM APO or VER with or without 20 µM enzalutamide, total cell number was determined at 3 days. (**c**) CWR22Rv1 cells were treated with 5 µM APO or VER with or without enzalutamide and abiraterone, the colonogenic assay was performed and colonies were quantified. * $p<0.05$. Results are the mean of three independent experiments (±S.D.). Statistical analysis was performed using one way ANOVA. ENZA: Enzalutamide, AA, Abiraterone acetate, APO: Apoptozole, VER: Ver155008.

Supplementary Figure 6

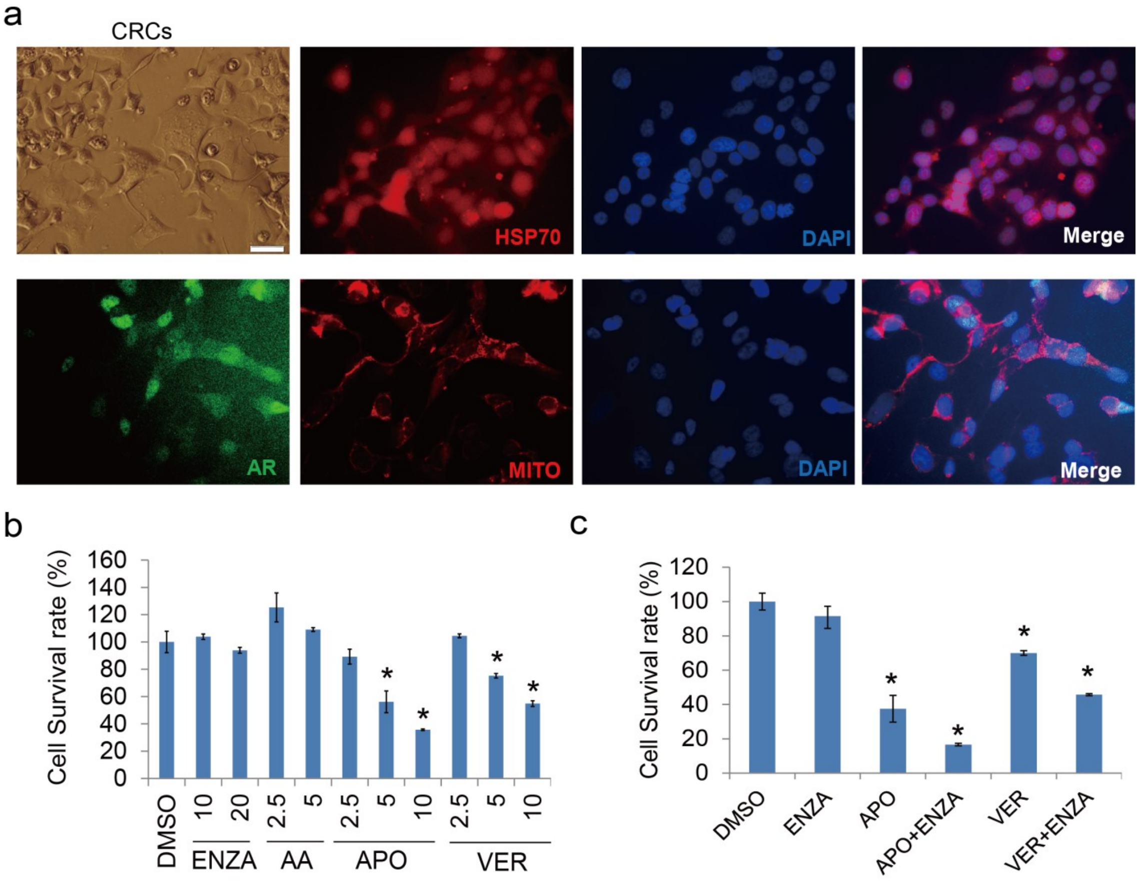

Supplementary Figure 6. HSP70 inhibitors suppress the growth of patient derived CRCs (a) Tumor tissues from Gleason 10 prostate cancer patient were processed and CRCs were established. HSP70 (F-3), AR (N20) and human mitochondria (MAB1273) were stained and visualized by fluorescence microscopy. Scale bar 20 μm. (**b**) Patient derived CRCs were treated with different doses of enzalutamide, abiraterone, APO and VER for 5 days, the cell survival rate was determined. (**c**) Patient derived CRCs were treated with 20μM enzalutamide with or without 10μM APO or VER for 5 days, the cell survival rate was determined. * p<0.05. Results are the mean of three independent experiments (±S.D.). Statistical analysis was performed using two tailed Student's t test. CRCs: Conditionally reprogrammed cells, ENZA: Enzalutamide, AA, Abiraterone acetate, APO: Apoptozole, VER: Ver155008, MITO: Mitochondrial.

Supplementary Figure 7

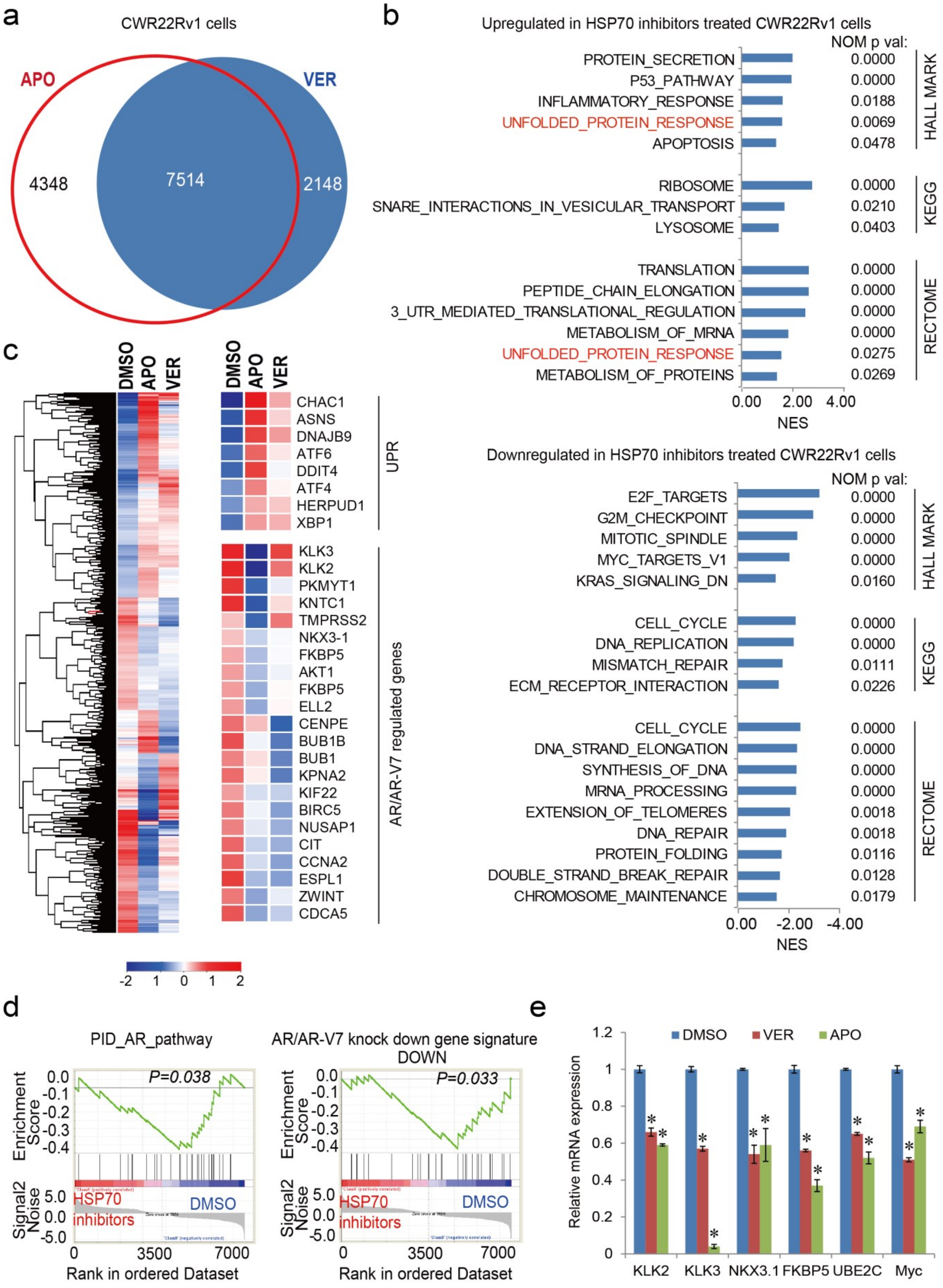

Supplementary Figure 7. HSP70 inhibitors block AR/AR-V7 pathways in CWR22Rv1 cells. (a) Venn diagram of RNA-seq analysis of APO vs DMSO and VER vs DMSO in CWR22Rv1 cells. **(b)** GSEA of top enriched gene sets in CWR22Rv1 cells treated by HSP70 inhibitors. The upregulated and down regulated gene sets from the Hallmark, KEGG and Rectome platforms were output by GSEA. **(c)** Heatmap and hierarchical clustering of the differentially expressed genes (DEGs) between APO and VER treatment in CWR22Rv1cells with FC > 1.2, as compared to vehicle (DMSO). The genes were displayed in rows and the normalized counts per sample were displayed in columns. Red indicates up-regulated and blue indicates down-regulated expression levels. Middle and right, UPR, AR and AR-V7 activity-signature genes that were altered in expression are displayed. **(d)** GSEA of the PID-AR pathway in CWR22Rv1 cells treated with HSP70 inhibitors, as compared to DMSO (left). GSEA of the AR and AR-V7 gene signatures in CWR22Rv1 cells treated with HSP70 inhibitors (right). The signature was defined by genes that underwent significant expression changes as a result of AR and AR-V7 knockdown in prostate cancer cells. **(e)** qRT-PCR analysis of the indicated genes in CWR22Rv1 cells treated with DMSO or with HSP70 inhibitors (10 μM) for 48 hours. *p<0.05. Results are the mean of three independent experiments (±S.D.). Statistical analysis was performed using two tailed Student's t test. APO: Apoptozole, VER: Ver155008.

Supplementary Figure 8

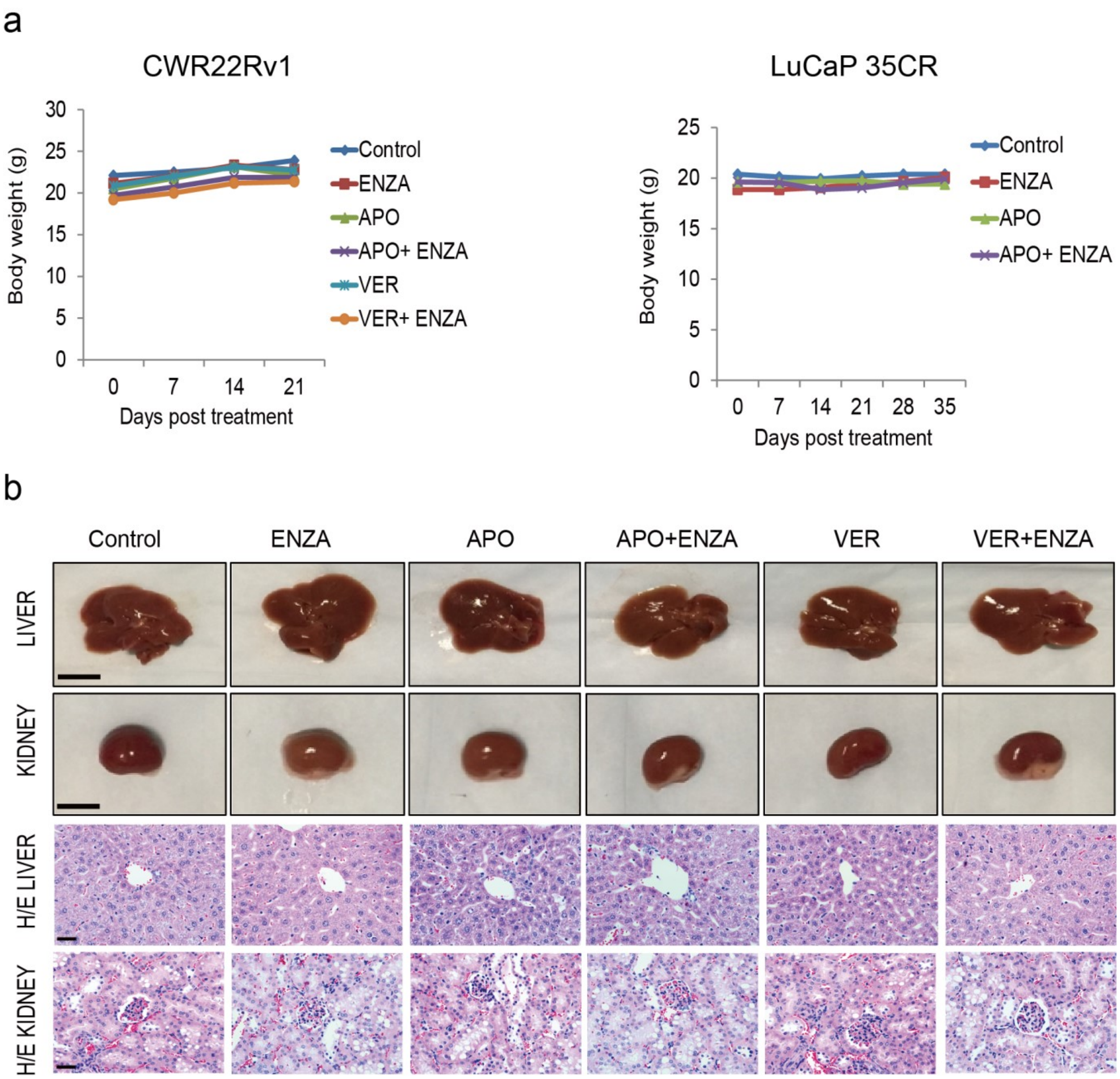

Supplementary Figure 8. APO and VER have no toxicity in mice. (a) Mice bearing CWR22Rv1 xenografts were treated with vehicle control, enzalutamide (25 mg per Kg p.o), APO (5 mg per Kg i.p), VER (15 mg per Kg i.p), APO plus enzalutamide and VER plus enzalutamide for 3 weeks. Body weights were determined weekly (left). Mice bearing LuCaP 35CR xenografts were treated with vehicle control, enzalutamide (25 mg per Kg p.o), APO (5 mg per Kg i.p) or their combination for 5 weeks. **(b)** Liver and kidney were harvested and photographed from CWR22Rv1 xenografts mice. Scale bar 1cm. H&E staining of liver, kidney and tumors from each group was performed. Scale bar 20 µm. ENZA: Enzalutamide, APO: Apoptozole, VER: Ver155008.

Supplementary Figure 9

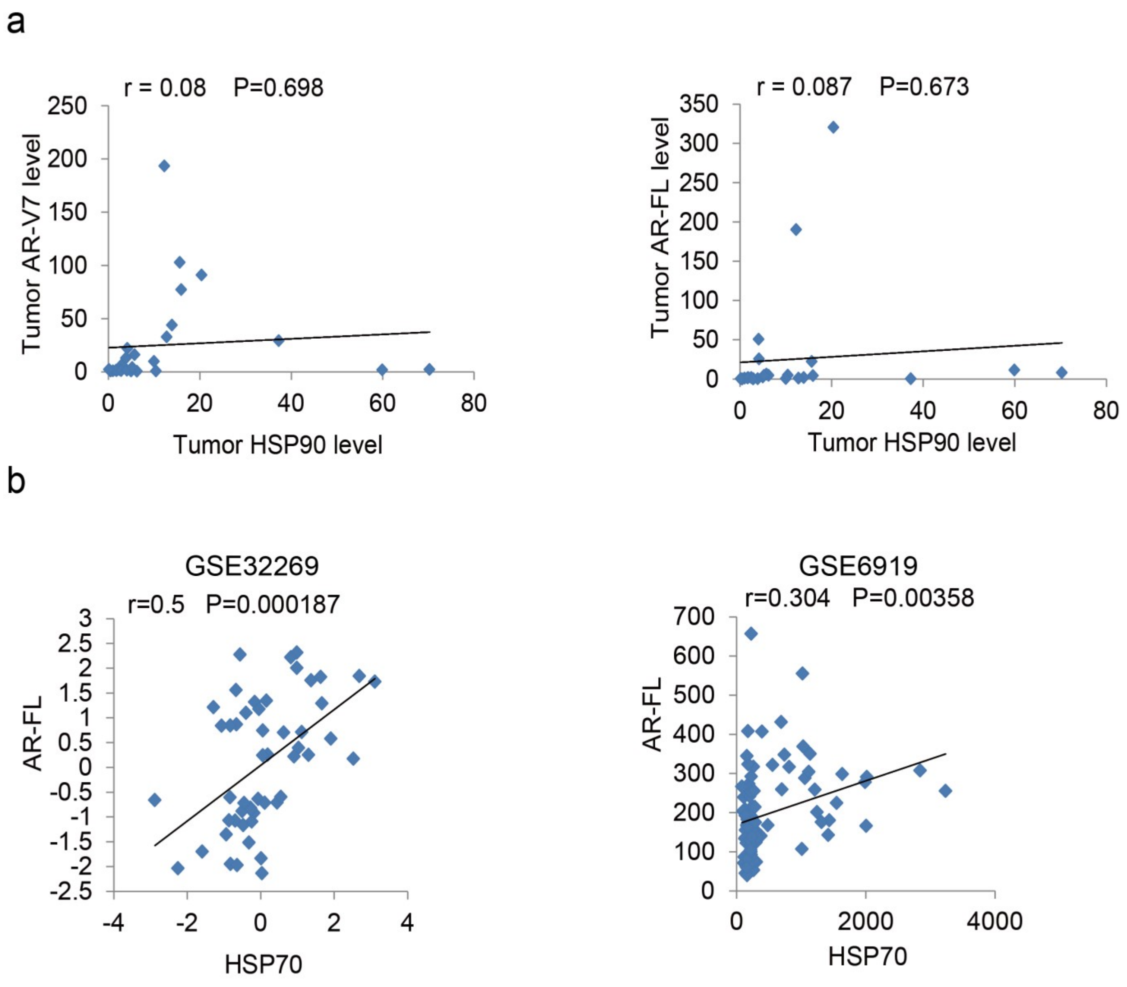

Supplementary Figure 9. The correlation of AR-FL/HSP90 and AR-V7/HSP90 in high Gleason score prostate tumors. Total RNA from 26 high Gleason score tumors was isolated and mRNA expression of AR-FL, AR-V7, HSP70 and HSP90 levels was determined. **(a)** AR-FL and HSP90 correlation (left), AR-V7 and HSP90 correlation (right) were determined by spearman rank correlation. **(b)** In two independent GEO data bases (GSE32269, GSE6919), AR and HSP70 gene expression were determined in primary prostate cancer (PCaP) and metastatic prostate cancer (MCaP) tumor samples. The AR-FL and HSP70 correlation was determined by spearman rank correlation.

Supplementary Fig.10

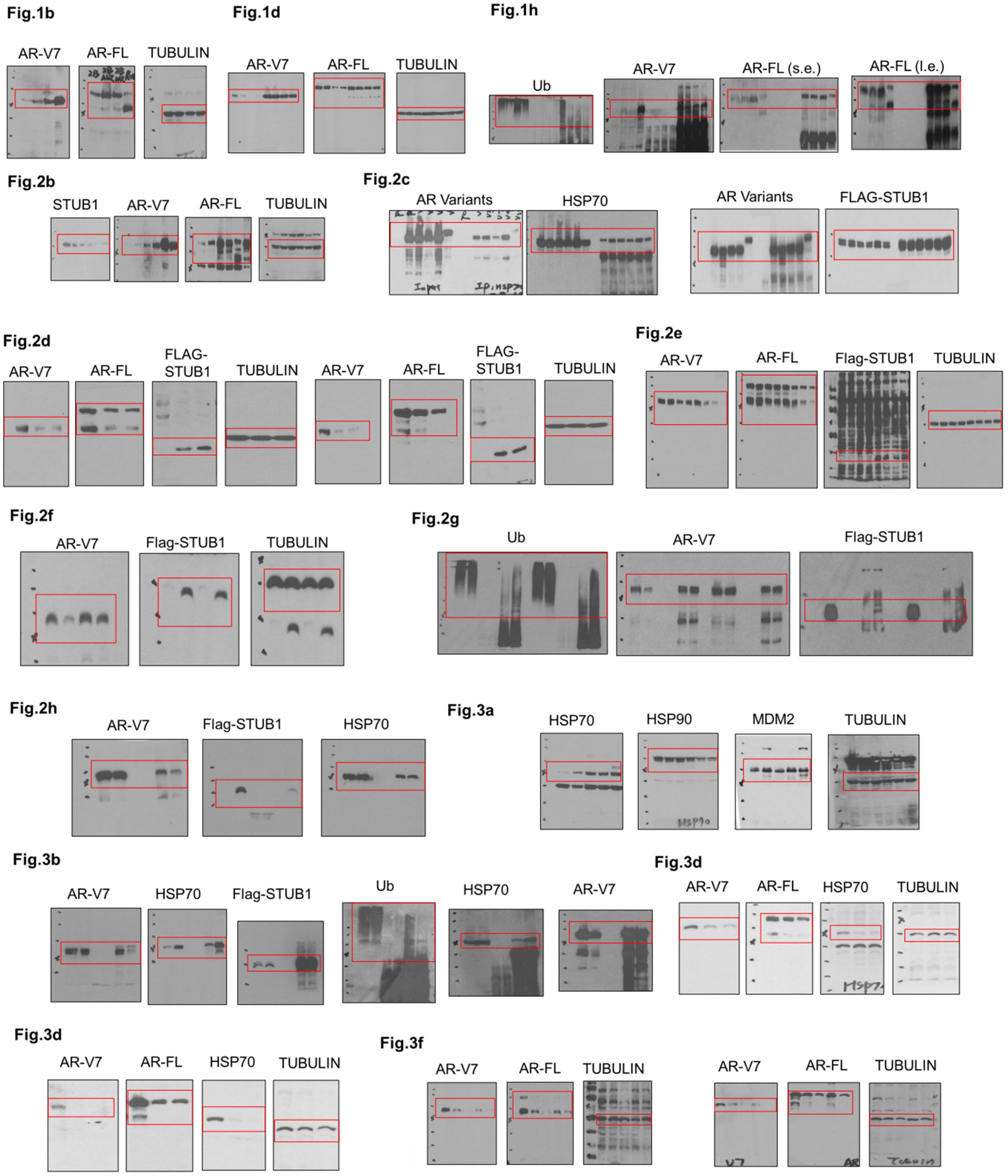

Supplementary Fig.11

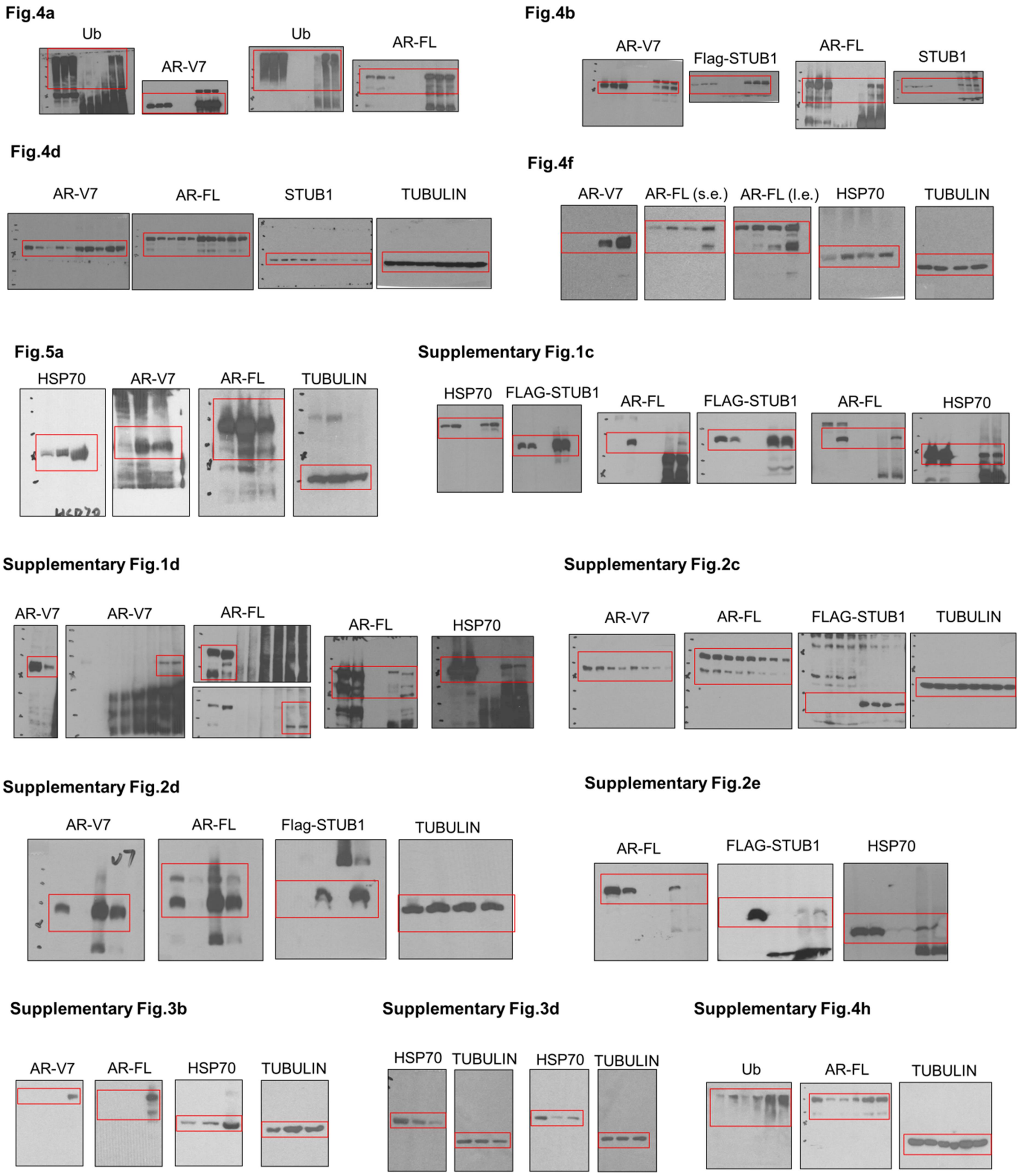

Supplementary Table 1. Patient information in the correlation study

UCD ID	ID	Biopsy site	Gleason Score	Tissue Type	Drug Treatment
**PS0085**	1	Prostate	10	HSPC*	None
**PS1168**	2	Prostate	9	CRPC#	Abiraterone, Enzalutamide
**PR0860**	3	Prostate	9	HSPC	None
**PS1169**	4	Prostate	10	CRPC	Lupron, Bicalutamide, Enzalutamide
**PR0911**	5	Prostate	9	HSPC	None
**PR0932**	6	Prostate	9	CRPC	SBRT Lilium
**PR0940**	7	Prostate	9	HSPC	None
**PR0967**	8	Prostate	8	HSPC	None
**PR0996**	9	Prostate	8	HSPC	None
**PS1153**	10	Prostate	8	HSPC	None
**PS0867**	11	Prostate	8	CRPC	Gosreilin, Bicalutamide
**PS0847**	12	Lymph nodes	9	CRPC	Bicalutamide
**PS0869**	13	Prostate	9	CRPC	Bicalutamide
**PS0895**	14	Prostate	8	CRPC	Flutamide
**PS1054**	15	Lymph nodes	9	CRPC	Bicalutamide
**PS574**	16	Prostate	8	HSPC	None
**PS457**	17	Prostate	8	HSPC	None
**PS608**	18	Prostate	8	HSPC	None
**20316**	19	Prostate	8	CRPC	Bicalutamide, Abiraterone, Enzalutamide
**PR1014**	20	Prostate	8	HSPC	None
**243-009**	21	Bone	9	CRPC	Leuprolide, Chemo
**PR1033**	22	Prostate	9	HSPC	None
**PS443-6**	23	Prostate	8	CRPC	Bicalutamide
**PS848**	24	Prostate	8	HSPC	None
**PS1171**	25	Bone	9	CRPC	Bicalutamide/Zoladex, Abiraterone, docetaxel, Enzalutamide
**PS1167**	26	Prostate	8	CRPC	Gosreilin, Bicalutamide

#CRPC: Castration resistant prostate cancer. *HSPC: Hormone sensitive prostate cancer

Supplementary Table 2. Up regulated gene sets in HSP70 inhibitors treated C4-2B MDVR cells

NAME	NES	NOM p-value
HALLMARK_UNFOLDED_PROTEIN_RESPONSE	2.24	0.0000
HALLMARK_P53_PATHWAY	1.98	0.0000
HALLMARK_PROTEIN_SECRETION	1.93	0.0022
HALLMARK_INFLAMMATORY_RESPONSE	1.88	0.0020
HALLMARK_COAGULATION	1.83	0.0041
HALLMARK_BILE_ACID_METABOLISM	1.76	0.0081
HALLMARK_HEME_METABOLISM	1.67	0.0043
HALLMARK_COMPLEMENT	1.56	0.0103
HALLMARK_XENOBIOTIC_METABOLISM	1.46	0.0402
HALLMARK_HYPOXIA	1.46	0.0289
KEGG_LYSOSOME	2.31	0.0000
KEGG_CELL_ADHESION_MOLECULES_CAMS	2.08	0.0000
KEGG_RIBOSOME	2.03	0.0062
KEGG_SNARE_INTERACTIONS_IN_VESICULAR_TRANSPORT	1.99	0.0020
KEGG_NATURAL_KILLER_CELL_MEDIATED_CYTOTOXICITY	1.90	0.0062
KEGG_CYTOKINE_CYTOKINE_RECEPTOR_INTERACTION	1.83	0.0041
KEGG_OXIDATIVE_PHOSPHORYLATION	1.73	0.0139
KEGG_SPHINGOLIPID_METABOLISM	1.70	0.0144
KEGG_ALANINE_ASPARTATE_AND_GLUTAMATE_METABOLISM	1.68	0.0199
KEGG_LEUKOCYTE_TRANSENDOTHELIAL_MIGRATION	1.64	0.0257
KEGG_N_GLYCAN_BIOSYNTHESIS	1.64	0.0201
REACTOME_SRP_DEPENDENT_COTRANSLATIONAL_PROTEIN_TARGETING_TO_MEMBRANE	2.48	0.0000
REACTOME_PEPTIDE_CHAIN_ELONGATION	2.33	0.0000
REACTOME_ANTIGEN_PRESENTATION_FOLDING_ASSEMBLY_AND_PEPTIDE_LOADING_OF_CLASS_I_MHC	2.10	0.0000
REACTOME_TRANSLATION	1.99	0.0000
REACTOME_ASPARAGINE_N_LINKED_GLYCOSYLATION	1.90	0.0041
REACTOME_CYTOSOLIC_TRNA_AMINOACYLATION	1.86	0.0040
REACTOME_IMMUNOREGULATORY_INTERACTIONS_BETWEEN_A_LYMPHOID_AND_A_NON_LYMPHOID_CELL	1.85	0.0020
REACTOME_UNFOLDED_PROTEIN_RESPONSE	1.80	0.0000
REACTOME_SPHINGOLIPID_METABOLISM	1.78	0.0063
REACTOME_INTERFERON_ALPHA_BETA_SIGNALING	1.77	0.0063
REACTOME_DIABETES_PATHWAYS	1.77	0.0041
REACTOME_INTERFERON_GAMMA_SIGNALING	1.76	0.0122
REACTOME_3_UTR_MEDIATED_TRANSLATIONAL_REGULATION	1.76	0.0000
REACTOME_POST_TRANSLATIONAL_PROTEIN_MODIFICATION	1.73	0.0040
REACTOME_ACTIVATION_OF_CHAPERONE_GENES_BY_XBP1S	1.65	0.0175
REACTOME_PHOSPHOLIPID_METABOLISM	1.64	0.0021
REACTOME_MEMBRANE_TRAFFICKING	1.64	0.0060
REACTOME_SPHINGOLIPID_DE_NOVO_BIOSYNTHESIS	1.64	0.0270
REACTOME_NONSENSE_MEDIATED_DECAY_ENHANCED_BY_THE_EXON_JUNCTION_COMPLEX	1.63	0.0020
REACTOME_TRANS_GOLGI_NETWORK_VESICLE_BUDDING	1.60	0.0252
REACTOME_AMINO_ACID_TRANSPORT_ACROSS_THE_PLASMA_MEMBRANE	1.60	0.0323
REACTOME_INFLUENZA_VIRAL_RNA_TRANSCRIPTION_AND_REPLICATION	1.57	0.0082
REACTOME_RESPIRATORY_ELECTRON_TRANSPORT	1.54	0.0432
REACTOME_ANTIGEN_PROCESSING_CROSS_PRESENTATION	1.53	0.0434

**Supplementary Table 2.** Cont'd		
REACTOME_ER_PHAGOSOME_PATHWAY	1.53	0.0285
REACTOME_TRANSPORT_OF_GLUCOSE_AND_OTHER_SUGARS_BILE_SALTS_AND_ORGANIC_ACIDS_METAL_IONS_AND_AMINE_COMPOUNDS	1.51	0.0342
REACTOME_METABOLISM_OF_AMINO_ACIDS_AND_DERIVATIVES	1.49	0.0166

Supplementary Table 3. Down regulated gene sets in HSP70 inhibitors treated C4-2B MDVR cells

NAME	NES	NOM p-value
HALLMARK_E2F_TARGETS	-3.86	0.0000
HALLMARK_G2M_CHECKPOINT	-3.63	0.0000
HALLMARK_MYC_TARGETS_V2	-2.96	0.0000
HALLMARK_MYC_TARGETS_V1	-2.74	0.0000
HALLMARK_MITOTIC_SPINDLE	-2.38	0.0000
HALLMARK_ANDROGEN_RESPONSE	-2.27	0.0000
HALLMARK_DNA_REPAIR	-1.61	0.0080
HALLMARK_SPERMATOGENESIS	-1.56	0.0369
KEGG_CELL_CYCLE	-2.99	0.0000
KEGG_DNA_REPLICATION	-2.90	0.0000
KEGG_SPLICEOSOME	-2.59	0.0000
KEGG_SYSTEMIC_LUPUS_ERYTHEMATOSUS	-2.23	0.0000
KEGG_HOMOLOGOUS_RECOMBINATION	-2.09	0.0000
KEGG_MISMATCH_REPAIR	-2.06	0.0020
KEGG_PYRIMIDINE_METABOLISM	-2.00	0.0000
KEGG_NUCLEOTIDE_EXCISION_REPAIR	-1.96	0.0000
KEGG_BASE_EXCISION_REPAIR	-1.85	0.0142
KEGG_RNA_DEGRADATION	-1.83	0.0059
KEGG_PURINE_METABOLISM	-1.83	0.0020
KEGG_OOCYTE_MEIOSIS	-1.72	0.0057
KEGG_PROGESTERONE_MEDIATED_OOCYTE_MATURATION	-1.69	0.0141
KEGG_PANCREATIC_CANCER	-1.63	0.0222
KEGG_COLORECTAL_CANCER	-1.55	0.0432
KEGG_SMALL_CELL_LUNG_CANCER	-1.55	0.0375
KEGG_PATHWAYS_IN_CANCER	-1.46	0.0134
REACTOME_CELL_CYCLE	-3.73	0.0000
REACTOME_CELL_CYCLE_MITOTIC	-3.60	0.0000
REACTOME_DNA_REPLICATION	-3.54	0.0000
REACTOME_S_PHASE	-3.49	0.0000
REACTOME_G1_S_TRANSITION	-3.48	0.0000
REACTOME_MITOTIC_G1_G1_S_PHASES	-3.45	0.0000
REACTOME_MITOTIC_M_M_G1_PHASES	-3.36	0.0000
REACTOME_SYNTHESIS_OF_DNA	-3.31	0.0000
REACTOME_DNA_STRAND_ELONGATION	-3.26	0.0000
REACTOME_E2F_MEDIATED_REGULATION_OF_DNA_REPLICATION	-3.18	0.0000
REACTOME_M_G1_TRANSITION	-3.13	0.0000
REACTOME_G1_S_SPECIFIC_TRANSCRIPTION	-3.09	0.0000
REACTOME_G2_M_CHECKPOINTS	-3.08	0.0000
REACTOME_CHROMOSOME_MAINTENANCE	-3.08	0.0000
REACTOME_ACTIVATION_OF_THE_PRE_REPLICATIVE_COMPLEX	-3.06	0.0000
REACTOME_PROCESSING_OF_CAPPED_INTRON_CONTAINING_PRE_ MRNA	-3.03	0.0000
REACTOME_TELOMERE_MAINTENANCE	-3.03	0.0000
REACTOME_MRNA_PROCESSING	-3.02	0.0000
REACTOME_ACTIVATION_OF_ATR_IN_RESPONSE_TO_REPLICATION_ STRESS	-3.00	0.0000
REACTOME_CELL_CYCLE_CHECKPOINTS	-2.89	0.0000
REACTOME_ASSEMBLY_OF_THE_PRE_REPLICATIVE_COMPLEX	-2.84	0.0000
REACTOME_MRNA_SPLICING	-2.80	0.0000
REACTOME_ORC1_REMOVAL_FROM_CHROMATIN	-2.75	0.0000
REACTOME_EXTENSION_OF_TELOMERES	-2.73	0.0000

Supplementary Table 3. Cont'd

REACTOME_TRANSCRIPTION	-2.68	0.0000
REACTOME_MITOTIC_PROMETAPHASE	-2.67	0.0000
REACTOME_G0_AND_EARLY_G1	-2.63	0.0000
REACTOME_DNA_REPAIR	-2.60	0.0000
REACTOME_MEIOSIS	-2.59	0.0000
REACTOME_MEIOTIC_RECOMBINATION	-2.58	0.0000
REACTOME_LAGGING_STRAND_SYNTHESIS	-2.57	0.0000
REACTOME_DEPOSITION_OF_NEW_CENPA_CONTAINING_NUCLEOSOMES_AT_THE_CENTROMERE	-2.47	0.0000
REACTOME_RNA_POL_I_RNA_POL_III_AND_MITOCHONDRIAL_TRANSCRIPTION	-2.47	0.0000
REACTOME_TRANSPORT_OF_MATURE_TRANSCRIPT_TO_CYTOPLASM	-2.46	0.0000
REACTOME_RNA_POL_I_TRANSCRIPTION	-2.40	0.0000
REACTOME_CLEAVAGE_OF_GROWING_TRANSCRIPT_IN_THE_TERMINATION_REGION_	-2.36	0.0000
REACTOME_METABOLISM_OF_NON_CODING_RNA	-2.31	0.0000
REACTOME_RNA_POL_II_TRANSCRIPTION	-2.30	0.0000
REACTOME_CYCLIN_E_ASSOCIATED_EVENTS_DURING_G1_S_TRANSITION_	-2.26	0.0000
REACTOME_HIV_LIFE_CYCLE	-2.24	0.0000
REACTOME_PACKAGING_OF_TELOMERE_ENDS	-2.24	0.0000
REACTOME_DOUBLE_STRAND_BREAK_REPAIR	-2.24	0.0000
REACTOME_NEP_NS2_INTERACTS_WITH_THE_CELLULAR_EXPORT_MACHINERY	-2.20	0.0000
REACTOME_TRANSPORT_OF_RIBONUCLEOPROTEINS_INTO_THE_HOST_NUCLEUS	-2.19	0.0000
REACTOME_MEIOTIC_SYNAPSIS	-2.19	0.0000
REACTOME_RNA_POL_I_PROMOTER_OPENING	-2.18	0.0000
REACTOME_MRNA_SPLICING_MINOR_PATHWAY	-2.16	0.0000
REACTOME_MITOTIC_G2_G2_M_PHASES	-2.15	0.0000
REACTOME_TRANSPORT_OF_MATURE_MRNA_DERIVED_FROM_AN_INTRONLESS_TRANSCRIPT	-2.14	0.0020
REACTOME_REGULATION_OF_GLUCOKINASE_BY_GLUCOKINASE_REGULATORY_PROTEIN	-2.14	0.0000
REACTOME_GLUCOSE_TRANSPORT	-2.13	0.0000
REACTOME_MRNA_3_END_PROCESSING	-2.12	0.0000
REACTOME_INTERACTIONS_OF_VPR_WITH_HOST_CELLULAR_PROTEINS	-2.09	0.0000
REACTOME_TRANSCRIPTION_COUPLED_NER_TC_NER	-2.08	0.0037
REACTOME_LATE_PHASE_OF_HIV_LIFE_CYCLE	-2.08	0.0000
REACTOME_AMYLOIDS	-2.07	0.0019
REACTOME_METABOLISM_OF_NUCLEOTIDES	-2.06	0.0000
REACTOME_GLOBAL_GENOMIC_NER_GG_NER	-2.02	0.0021
REACTOME_REGULATION_OF_MITOTIC_CELL_CYCLE	-2.00	0.0020
REACTOME_ANTIVIRAL_MECHANISM_BY_IFN_STIMULATED_GENES	-1.97	0.0019
REACTOME_NUCLEOTIDE_EXCISION_REPAIR	-1.96	0.0038
REACTOME_FORMATION_OF_TUBULIN_FOLDING_INTERMEDIATES_BY_CCT_TRIC	-1.92	0.0019
REACTOME_DEADENYLATION_DEPENDENT_MRNA_DECAY	-1.89	0.0078
REACTOME_GLYCOLYSIS	-1.89	0.0097
REACTOME_SCFSKP2_MEDIATED_DEGRADATION_OF_P27_P21	-1.89	0.0055
REACTOME_PURINE_METABOLISM	-1.86	0.0093
REACTOME_RNA_POL_III_TRANSCRIPTION	-1.84	0.0059

Supplementary Table 3. Cont'd

REACTOME_G1_PHASE	-1.82	0.0075
REACTOME_HIV_INFECTION	-1.80	0.0000
REACTOME_METABOLISM_OF_RNA	-1.77	0.0036
REACTOME_CDT1_ASSOCIATION_WITH_THE_CDC6_ORC_ORIGIN_COMPLEX	-1.75	0.0118
REACTOME_LOSS_OF_NLP_FROM_MITOTIC_CENTROSOMES	-1.72	0.0119
REACTOME_PREFOLDIN_MEDIATED_TRANSFER_OF_SUBSTRATE_TO_CCT_TRIC	-1.69	0.0223
REACTOME_HOST_INTERACTIONS_OF_HIV_FACTORS	-1.66	0.0222
REACTOME_RECRUITMENT_OF_MITOTIC_CENTROSOME_PROTEINS_AND_COMPLEXES	-1.64	0.0248
REACTOME_FACTORS_INVOLVED_IN_MEGAKARYOCYTE_DEVELOPMENT_AND_PLATELET_PRODUCTION	-1.62	0.0080
REACTOME_SIGNALING_BY_RHO_GTPASES	-1.58	0.0261
REACTOME_RNA_POL_I_TRANSCRIPTION_INITIATION	-1.59	0.0325
REACTOME_HOST_INTERACTIONS_OF_HIV_FACTORS	-1.57	0.0160
REACTOME_MRNA_CAPPING	-1.55	0.0317
REACTOME_CDT1_ASSOCIATION_WITH_THE_CDC6_ORC_ORIGIN_COMPLEX	-1.55	0.0282
REACTOME_GLUCOSE_METABOLISM	-1.54	0.0254
REACTOME_RNA_POL_II_TRANSCRIPTION_PRE_INITIATION_AND_PROMOTER_OPENING	-1.51	0.0430
REACTOME_RNA_POL_III_TRANSCRIPTION_TERMINATION	-1.50	0.0500
REACTOME_PROTEIN_FOLDING	-1.49	0.0460
REACTOME_SIGNALING_BY_RHO_GTPASES	-1.48	0.0271
REACTOME_RNA_POL_II_PRE_TRANSCRIPTION_EVENTS	-1.43	0.0477

Supplementary Table 4. Up regulated gene sets in HSP70 inhibitors treated CWR22Rv1 cells

**NAME**	**NES**	**NOM p-value**
HALLMARK_PROTEIN_SECRETION	2.00	0.0000
HALLMARK_P53_PATHWAY	1.97	0.0000
HALLMARK_HEME_METABOLISM	1.78	0.0000
HALLMARK_INFLAMMATORY_RESPONSE	1.62	0.0188
HALLMARK_UNFOLDED_PROTEIN_RESPONSE	1.60	0.0069
HALLMARK_TNFA_SIGNALING_VIA_NFKB	1.45	0.0326
HALLMARK_APOPTOSIS	1.36	0.0478
KEGG_RIBOSOME	2.77	0.0000
KEGG_SNARE_INTERACTIONS_IN_VESICULAR_TRANSPORT	1.69	0.0210
KEGG_LYSOSOME	1.46	0.0403
REACTOME_SRP_DEPENDENT_COTRANSLATIONAL_PROTEIN_TARGETING_TO_MEMBRANE	2.79	0.0000
REACTOME_TRANSLATION	2.64	0.0000
REACTOME_PEPTIDE_CHAIN_ELONGATION	2.64	0.0000
REACTOME_3_UTR_MEDIATED_TRANSLATIONAL_REGULATION	2.51	0.0000
REACTOME_NONSENSE_MEDIATED_DECAY_ENHANCED_BY_THE_EXON_JUNCTION_COMPLEX	2.42	0.0000
REACTOME_INFLUENZA_VIRAL_RNA_TRANSCRIPTION_AND_REPLICATION	2.38	0.0000
REACTOME_FORMATION_OF_THE_TERNARY_COMPLEX_AND_SUBSEQUENTLY_THE_43S_COMPLEX	2.06	0.0021
REACTOME_ACTIVATION_OF_THE_MRNA_UPON_BINDING_OF_THE_CAP_ BINDING_COMPLEX_AND_EIFS_AND_SUBSEQUENT_BINDING_TO_43S	1.94	0.0023
REACTOME_GENERIC_TRANSCRIPTION_PATHWAY	1.90	0.0000
REACTOME_METABOLISM_OF_MRNA	1.84	0.0000
REACTOME_INFLUENZA_LIFE_CYCLE	1.72	0.0065
REACTOME_UNFOLDED_PROTEIN_RESPONSE	1.57	0.0275
REACTOME_PACKAGING_OF_TELOMERE_ENDS	1.56	0.0418
REACTOME_AMINO_ACID_TRANSPORT_ACROSS_THE_PLASMA_MEMBRANE	1.55	0.0498
REACTOME_DIABETES_PATHWAYS	1.55	0.0179
REACTOME_SIGNALING_BY_ERBB4	1.52	0.0470
REACTOME_METABOLISM_OF_PROTEINS	1.40	0.0269

Supplementary Table 5. Down regulated gene sets in HSP70 inhibitors treated CWR22Rv1 cell

NAME	NES	NOM p-value
HALLMARK_E2F_TARGETS	-3.24	0.0000
HALLMARK_G2M_CHECKPOINT	-2.99	0.0000
HALLMARK_MYC_TARGETS_V2	-2.51	0.0000
HALLMARK_MITOTIC_SPINDLE	-2.37	0.0000
HALLMARK_MYC_TARGETS_V1	-2.05	0.0000
HALLMARK_ESTROGEN_RESPONSE_LATE	-1.63	0.0053
HALLMARK_KRAS_SIGNALING_DN	-1.50	0.0160
HALLMARK_SPERMATOGENESIS	-1.50	0.0495
KEGG_CELL_CYCLE	-2.29	0.0000
KEGG_DNA_REPLICATION	-2.22	0.0000
KEGG_PROGESTERONE_MEDIATED_OOCYTE_MATURATION	-2.09	0.0038
KEGG_FC_GAMMA_R_MEDIATED_PHAGOCYTOSIS	-1.92	0.0018
KEGG_PURINE_METABOLISM	-1.91	0.0054
KEGG_PATHOGENIC_ESCHERICHIA_COLI_INFECTION	-1.85	0.0056
KEGG_GAP_JUNCTION	-1.84	0.0053
KEGG_BASE_EXCISION_REPAIR	-1.81	0.0056
KEGG_PYRIMIDINE_METABOLISM	-1.80	0.0018
KEGG_MISMATCH_REPAIR	-1.78	0.0111
KEGG_HOMOLOGOUS_RECOMBINATION	-1.78	0.0038
KEGG_GLYCEROLIPID_METABOLISM	-1.73	0.0230
KEGG_OOCYTE_MEIOSIS	-1.70	0.0147
KEGG_ECM_RECEPTOR_INTERACTION	-1.63	0.0226
KEGG_SMALL_CELL_LUNG_CANCER	-1.55	0.0289
KEGG_DILATED_CARDIOMYOPATHY	-1.52	0.0499
REACTOME_CELL_CYCLE_MITOTIC	-2.83	0.0000
REACTOME_DNA_REPLICATION	-2.61	0.0000
REACTOME_MITOTIC_M_M_G1_PHASES	-2.61	0.0000
REACTOME_MITOTIC_G1_G1_S_PHASES	-2.60	0.0000
REACTOME_G1_S_TRANSITION	-2.55	0.0000
REACTOME_ACTIVATION_OF_THE_PRE_REPLICATIVE_COMPLEX	-2.53	0.0000
REACTOME_S_PHASE	-2.49	0.0000
REACTOME_CELL_CYCLE	-2.49	0.0000
REACTOME_G1_S_SPECIFIC_TRANSCRIPTION	-2.43	0.0000
REACTOME_G2_M_CHECKPOINTS	-2.42	0.0000
REACTOME_PROCESSING_OF_CAPPED_INTRON_CONTAINING_PRE_MRNA	-2.38	0.0000
REACTOME_ACTIVATION_OF_ATR_IN_RESPONSE_TO_REPLICATION_STRESS	-2.36	0.0000
REACTOME_POTASSIUM_CHANNELS	-2.36	0.0000
REACTOME_M_G1_TRANSITION	-2.36	0.0000
REACTOME_DNA_STRAND_ELONGATION	-2.35	0.0000
REACTOME_SYNTHESIS_OF_DNA	-2.34	0.0000

Supplementary Table 5. Cont'd

REACTOME_MRNA_PROCESSING	-2.32	0.0000
REACTOME_E2F_MEDIATED_REGULATION_OF_DNA_REPLICATION	-2.30	0.0000
REACTOME_ASSEMBLY_OF_THE_PRE_REPLICATIVE_COMPLEX	-2.24	0.0000
REACTOME_MITOTIC_G2_G2_M_PHASES	-2.24	0.0000
REACTOME_MRNA_SPLICING	-2.16	0.0000
REACTOME_MITOTIC_PROMETAPHASE	-2.11	0.0000
REACTOME_EXTENSION_OF_TELOMERES	-2.08	0.0018
REACTOME_ORC1_REMOVAL_FROM_CHROMATIN	-2.03	0.0000
REACTOME_OPIOID_SIGNALLING	-2.01	0.0000
REACTOME_LOSS_OF_NLP_FROM_MITOTIC_CENTROSOMES	-1.99	0.0020
REACTOME_METABOLISM_OF_VITAMINS_AND_COFACTORS	-1.99	0.0018
REACTOME_CELL_CYCLE_CHECKPOINTS	-1.98	0.0000
REACTOME_METABOLISM_OF_NON_CODING_RNA	-1.96	0.0000
REACTOME_LAGGING_STRAND_SYNTHESIS	-1.94	0.0000
REACTOME_DNA_REPAIR	-1.92	0.0018
REACTOME_GPCR_LIGAND_BINDING	-1.88	0.0017
REACTOME_SIGNALING_BY_GPCR	-1.84	0.0000
REACTOME_RECRUITMENT_OF_MITOTIC_CENTROSOME_PROTEINS_AND_COMPLEXES	-1.84	0.0037
REACTOME_G_ALPHA_Q_SIGNALLING_EVENTS	-1.83	0.0038
REACTOME_GLYCOLYSIS	-1.82	0.0019
REACTOME_CLASS_A1_RHODOPSIN_LIKE_RECEPTORS	-1.82	0.0054
REACTOME_PLC_BETA_MEDIATED_EVENTS	-1.81	0.0054
REACTOME_METABOLISM_OF_CARBOHYDRATES	-1.80	0.0000
REACTOME_PHOSPHOLIPASE_C_MEDIATED_CASCADE	-1.80	0.0058
REACTOME_GASTRIN_CREB_SIGNALLING_PATHWAY_VIA_PKC_AND_MAPK	-1.78	0.0074
REACTOME_PROCESSIVE_SYNTHESIS_ON_THE_LAGGING_STRAND	-1.77	0.0018
REACTOME_TRANSPORT_OF_MATURE_TRANSCRIPT_TO_CYTOPLASM	-1.77	0.0133
REACTOME_PROTEIN_FOLDING	-1.74	0.0116
REACTOME_PLATELET_ACTIVATION_SIGNALING_AND_AGGREGATION	-1.74	0.0035
REACTOME_GPCR_DOWNSTREAM_SIGNALING	-1.71	0.0019
REACTOME_DOUBLE_STRAND_BREAK_REPAIR	-1.68	0.0128
REACTOME_G_ALPHA_I_SIGNALLING_EVENTS	-1.65	0.0133
REACTOME_METABOLISM_OF_NUCLEOTIDES	-1.64	0.0184
REACTOME_GLUCOSE_METABOLISM	-1.61	0.0166
REACTOME_NEURONAL_SYSTEM	-1.61	0.0051
REACTOME_CHROMOSOME_MAINTENANCE	-1.55	0.0179
REACTOME_HIV_LIFE_CYCLE	-1.55	0.0369
REACTOME_PEPTIDE_LIGAND_BINDING_RECEPTORS	-1.50	0.0418
REACTOME_INTEGRATION_OF_ENERGY_METABOLISM	-1.49	0.0471
REACTOME_PLATELET_HOMEOSTASIS	-1.49	0.0499
REACTOME_TRANSCRIPTION_COUPLED_NER_TC_NER	-1.49	0.0526
REACTOME_G_ALPHA_Z_SIGNALLING_EVENTS	-1.48	0.0693
REACTOME_HEMOSTASIS	-1.48	0.0103

Supplementary Table 6. Primer list for the qRT-PCR

	Forward	Reverse
AR-V1	AACAGAAGTACCTGTGCGCC	TGAGACTCCAAACACCCTCA
AR-V3	TGGATGGATAGCTACTCCGG	GTTCATTCTGAAAAATCCTTCAGC
AR-V4	AACAGAAGTACCTGTGCGCC	TTCTGTCAGTCCCATTGGTG
AR-V7	AACAGAAGTACCTGTGCGCC	TCAGGGTCTGGTCATTTTGA
AR-V9	TGCGCCAGCAGAAATGATTG	GCAGCTGCTCAGGTAAGTTG
AR-FL	AAGCCAGAGCTGTGCAGATGA	TGTCCTGCAGCCACTGGTTC
HSP70 (HSPA1B)	TGGACTGTTGGGACTCAAGGAC	GGAACGAAACACCCTTACAGTATCA
HSP90 (HSP90AA1)	AGCTCAAGCCCTAAGAGACAACT	AAGATGACCAGATCCTTCACAGA
STUB1	AGGCCAAGCACGACAAGTACAT	CTGATCTTGCCACACAGGTAGT
KLK2	CAACATCTGGAGGGGAAAGGG	AGGCCAAGTGATGCCAGAAC
KLK3	GCCCTGCCCGAAAGG	GATCCACTTCCGGTAATGCA
FKBP5	GGGAAGATAGTGTCCTGGTTAG	GCAGTCTTGCAGCCTTATTC
NKX3-1	CCGAGACGCTGGCAGAGACC	GCTTAGGGGTTTGGGGAAG
UBE2C	TGGTCTGCCCTGTATGATGT	AAAAGCTGTGGG GTTTTTCC
Myc	TGAGGAGACACCGCCCAC	CAACATCGATTTCTTCCTCATC
ACTIN	AGAACTGGCCCTTCTTGGAGG	GTTTTTATGTTCCTCTATGGG
